# Spatial inequalities in charitable fundraising and income generation for NHS acute trusts in England

**DOI:** 10.1080/09540962.2024.2304541

**Published:** 2024-02-09

**Authors:** John Mohan, David Clifford

**Affiliations:** a Third Sector Research Centre, University of Birmingham, UK; b University of Southampton, UK

**Keywords:** Charitable income, local area deprivation, NHS trusts, private patient income, spatial inequality

## Abstract

**IMPACT:**

This article presents novel analyses of the income sources of National Health Service (NHS) acute trusts in England. The results suggest that there are variations according to deprivation in the extent of private financial resources available to NHS institutions. They suggest a need to open up discussions about how best to mitigate spatial differences in the charitable and private patient income of NHS trusts, particularly if these sources of income grow in importance going forward.

**ABSTRACT:**

The article provides—for the first time—an analysis of spatial variation in the income sources of National Health Service (NHS) acute trusts in England. It shows that, compared to trusts serving less deprived communities, trusts serving more deprived communities receive a lower proportion of income from charitable sources; and that trusts serving deprived communities also receive a lower proportion of income from private patients. The study serves to provide evidence of spatial inequality in the private resources that support local public institutions.

## Introduction: area deprivation and the institutional context of public service provision

Public services in the United Kingdom, such as the National Health Service (NHS), are characterized by universalist aspirations and a high degree of centralization of finance. A key aim of NHS policy was to eliminate the inequalities in the pattern of services inherited at the establishment of the service in 1948. Various central government initiatives aspired substantially to reduce spatial inequalities in provision through central planning of capital and revenue funding allocations. These initiatives were driven by assessments of the relative needs of geographical areas and were designed to compensate for patterns of disadvantage, and there are good reasons why those patterns would be the target of policy interventions. Local area deprivation is seen as a problem in the context of discussions about ‘area effects’, whereby local areas of deprivation (which may be at a larger spatial scale than neighbourhoods) ‘are seen adversely to affect life chances above and beyond individual characteristics’ (Rae, 2012, p. 1184).

However, within social policy a key challenge is to identify *processes* through which area deprivation is mediated. Important theory argues that, if ‘local area effects’ exist—whereby the level of area deprivation has an independent effect on individual wellbeing—presumably they stem from ‘collective’ properties of local areas (Sampson, 2012, p. 47). One such collective property may be the differences in institutional resources between more and less deprived local areas (Galster, 2012; Reich, 2018).

Providing empirical evidence to test this institutional resources perspective has proved a challenge and remains a key research priority (Sampson, 2012; Small & McDermott, 2006; Sharkey & Faber, 2014; Small, 2014). In this article, we extend this perspective by arguing that, while the NHS has to a degree eliminated inequalities in resources, there are variations in the extent to which local resources, differentially available to institutions, may work against the grain of redistributive policies.

In a universal service which is funded and planned centrally, the basis for a negative link between local area deprivation and the institutional context of public service provision is not intuitive (Hastings, 2009; Galster, 2012). However, as Hastings (2009, p. 509) argues, there is a need to draw out the ways in which the nature of, and resources for, public service provision can be affected by area disadvantage even in situations where the ‘welfare state regime is sufficiently robust’ that more deprived areas ‘retain a significant public institutional infrastructure’. As examples, Hastings (2009) points to Lupton’s (2004) research on the difficulties that more deprived areas face in recruiting quality teachers for schools, and Mackay et al.’s (2005) research on the difficulties that more deprived areas face in recruiting quality general medical practitioners.

In this regard, and at the risk of oversimplifying a complex history, from the election of the Thatcher government in 1979, the NHS experienced a succession of reforms which removed various restrictions on the capacity of the local operational units of the service to generate and retain resources. (Note that, while the ‘Big Society’ idea in the 2010 UK Conservative party’s general election manifesto may have failed to achieve political traction, it served to reiterate—rhetorically if not in practice—this 1980s policy thrust about opening up models of public funding.)

In this article we therefore examine the extent to which spatial variations in deprivation may affect the ability of individual English NHS trusts to supplement their financial resources from government with voluntary income, through charitable fundraising and income generation, through income from private patients.

## Theoretical rationale: spatial manifestations of resource insufficiency

Almost all healthcare systems permit some degree of fee-for-service provision of care, funded through direct payments from individuals, or through private insurance schemes. The connection with local socioeconomic context is therefore self-evident: the more prosperous the locality, and therefore the higher the average level of income, the more likely it is that individuals will be able to access private treatment. We also know that healthcare insurance policies in the UK are largely funded by employers and that, historically, the recipients of these policies are most likely to reside in the most prosperous communities. Therefore we would anticipate that NHS trusts located in such places would be able to leverage more funding through the treatment of private patients.

Salamon’s (1987) theory of voluntary sector failure provides a basis for expecting local area differences in voluntary income through charitable fundraising. Salamon argues that voluntarism cannot generate resources that can adequately and reliably ‘cope with the human services problems of an advanced industrial society’ (p. 39). Importantly, this ‘resource insufficiency’ has spatial manifestations, ‘since the resources are frequently not available where the problems are most severe’ (p. 40). Opportunities for organizations to benefit from private income, including both voluntary donations and private fees from individuals, may be very different in different kinds of areas. ‘Serious gaps’ in coverage may emerge because ‘private [charitable and fee income] resources may or may not be available where the need for them is greatest’ (p. 45). Therefore, when considering the implications for local organizational activity, this focuses attention not just on the demand for public goods and services (Weisbrod, 1975) but also on spatial variations in the supply of resources. In deprived areas, where people may struggle to meet basic needs, combining internal resources ‘can represent little more than multiplying zero times zero; the result is still zero’ (Logan & Molotch, 1987, pp. 136–137).

Indeed, the relationship between financial resources and support for charities is clear: those with more financial means give higher amounts (Bekkers & Wiepking, 2006; Wiepking & Bekkers, 2012). Level of education is also a key individual predictor of charitable giving: those with higher levels of education—who may be more aware of social need, more exposed to information about charitable causes and more likely to be requested to donate—give more (Bekkers & Wiepking, 2011). Therefore there is a theoretical basis for expecting a link between the ‘compositional’ characteristics of local areas, in terms of the financial and educational resources of their residents, and an important collective or ‘emergent’ characteristic: the aggregate level of charitable giving in a community. Thus local areas that differ in levels of deprivation may differ in terms of their ability to provide the financial resources for local institutions (Salamon, 1987). The concomitant potential for unevenness in voluntary organizational activity was emphasised by the influential Wolfenden Report, which argued that ‘some social and geographical contexts seem to provide a much more fertile soil for [voluntary] action than others’ (Wolfenden, 1978, p. 58; see also JRF, 2011). Indeed, considerable community-level inequalities in voluntary hospital provision in England were well-documented from the late 19th century onwards (Mohan, 2002). The financing of these voluntary hospitals, predominantly through charitable support, led to considerable variations between communities in terms of hospital capacity and expenditure (Mohan & Gorsky, 2001).

While Salamon’s (1987) theory was developed in relation to voluntary organizations, its insights are relevant more generally to organizations providing goods and services that draw—even for a minority of their income—on private sources of funding. For example, Reich (2018, p. 96) argues that local education foundations, which are linked to schools and school districts in the USA, and which raise private money to supplement the public funding of schooling, exacerbate inequalities since ‘wealthy schools and school districts can raise substantially more money than can schools that have high concentrations of poor students’.

In this article, we develop this interest in spatial variations in private funding for public services by assessing the spatial manifestations of resource insufficiency for NHS trusts in England. According to the level of deprivation, local areas may differ not only in the opportunity for trusts to benefit from charitable donations, but also in the opportunity for trusts to receive income from private patients. NHS trusts in more deprived local areas may be expected to be *doubly disadvantaged* since, compared to their counterparts in more prosperous areas, they are less able to supplement public funding with income from individuals’ charitable donations and private fees (Salamon, 1987; Clifford et al., 2013).

## Substantive context: charitable fundraising and income generation in the NHS

Here we outline the processes through which liberalization of the NHS has taken place over the past four decades. The cumulative result of these processes has been the expansion of the scope for the operational units of the service to generate income from a range of sources.

### Voluntary income through charitable fundraising

While the NHS is perceived as being a state-funded and state-run healthcare system, charitable sources of funding for healthcare did not disappear following the 1948 founding legislation. The receipt of charitable donations was always permitted (Fitzherbert, 1989), but fundraising by NHS authorities was prohibited until 1980 when, through the 1980 Health Services Act, the Conservative government empowered health authorities to organize their own charitable appeals. This development can be seen as an element of Conservative strategy to expand the scope for non-statutory funding of health and welfare services (Mohan, 1995). In an international context we may see it as part of a wider set of processes of the restructuring of healthcare in which formerly-integrated publicly-financed healthcare systems are disaggregated into units which compete against one another for resources (Griffith et al., 1987; Salmon, 1995; Brown & Barnett, 2004; Leys, 2001; Mohan, 2002; Pollock, 2004).

Market liberalization also took the form of granting the key operational units of the NHS greater autonomy—initially through the 1991 reforms of the service, which established the ‘purchaser–provider split’ in which ‘purchasers’ of healthcare arranged contracts with providers of services—principally NHS trusts (characteristically combinations of multiple providers of care). Subsequent reforms in 2003 legislated for NHS foundation trusts which, for the first time since 1948, were no longer accountable directly to government. Today, the provision of NHS treatment is substantially delivered through 217 NHS trusts (in England), most of which have achieved foundation status. They compete for contracts to deliver NHS services. NHS trusts vary considerably in size and complexity; they include providers of acute hospital care, ambulance and patient transport services, mental health and community health services, and highly specialized services for specific health conditions. Each trust is associated with an NHS charity—an entity established for charitable purposes related to the NHS, which receives charitable income. (Note that charitable status—the primary legal framework for voluntary activity in the UK—is underpinned by the criterion of ‘public benefit’ — that an organization should ‘benefit the public in general or a significant section of the public’.)

Collectively NHS charities give £1 million every day to support the NHS (NPC, 2019). Officially charitable funds are ‘supplementary’ to the resources provided by government and there is a convention, though not one codified in law, that these funds should not be used to substitute those provided by the government for the delivery of NHS provision. This is a difficult border to demarcate with precision; in practice, charitable funds are put to a range of purposes, encompassing the immediate practical and emotional needs of staff and patients, the purchase of medical equipment, the funding of research and development, and improvements to hospital environments (Bowles et al., 2023; NHS Charities Together, 2022).

Existing studies of charitable giving to the NHS provide estimates of aggregate levels of income and expenditure, highlighting some organizations with particularly large charitable funds, and identifying ways in which the uneven distribution of charitable resources poses challenges for NHS authorities (Fitzherbert, 1989; Lattimer & Holly, 1992; Lattimer et al., 1996; Mohan & Gorsky, 2001; Pharoah & Mocroft, 2001; Exworthy & Lafond, 2021). However, importantly, to date there has been limited collective understanding about variations in the extent to which different kinds of NHS trusts benefit from charitable funding; specifically, there is an absence of work on spatial variations in charitable fundraising between different trusts. For example, what is the relationship between charitable income and total income across the population of NHS trusts; how unevenly distributed is it; and how does it relate to social conditions in the areas in which trusts are located? These are the questions we explore here. In a complementary paper we examine differences in charitable funding according to the sector of the trust (acute/ambulance/community/mental health/specialist) (Bowles et al., 2023).

### Income generation through private patient activity

NHS treatment has always been free at the point of use, but NHS hospitals have always been permitted to treat private fee-paying patients, and to retain surpluses generated by doing so. The treatment of private patients in NHS hospitals has at times attracted controversy, most notably in the 1970s. The 1974–1979 Labour government sought to abolish private beds in the NHS altogether; however, even then, there were concerns that the NHS ought not to reject large potential revenue streams (Castle, 1980). Since the early 1980s the UK’s NHS has been positively encouraged to seek to diversify its funding base and the expansion of private patient treatment formed part of a wider suite of entrepreneurial activities, such as income generation from property assets or by selling spare capacity or particular expertise (Mohan, 1995; Pollock, 2004). Policy guidance is that these activities must be run on a strictly commercial basis; they cannot be cross-subsidised from NHS budgets. There are estimates of the revenues thus generated, but not of the net surpluses available to NHS authorities. A succession of reforms designed to liberalize the NHS, and to remove constraints on the activities of the operational units of the service, saw discussion of whether to restrict the growth of fee-paying patients within the NHS. The establishment of NHS trusts in 1991, and particularly the creation of NHS foundation trusts in 2003, were both marked by criticisms of the greater potential for these entities to pursue or expand sources of income derived from market transactions. There was a debate in 2003 about the need for and level of a potential cap on NHS income from private patients, in response to concerns that NHS foundation trusts would prioritize institutional financial stability over the needs of NHS patients. The cap was constrained by historical levels of private income, so one institution, the Royal Marsden, had a cap fixed at around 30% of its total budget, though for most providers the figures were considerably smaller (Appleby, 2009). From 2012, further reforms to the NHS set the cap on non-NHS income for NHS foundation trusts at 49%: put another way, reflecting their public purposes, NHS trusts had to ensure that the majority of their funding came from the public purse.

If NHS trusts sought to expand their revenues from private patients, where would the patients come from? The majority of private patients in the UK who are British residents have insurance policies which cover the cost of inpatient treatment, but these people represent about one-eighth of the adult UK population and the numbers in possession of such policies have largely flatlined for some years now. However, significant regional variations have always been a feature of the insurance market. An important reason for this is that demand for private insurance is to a large part a ‘reflection of arrangements made by industry for key employees’ (Lee, 1978, p. 11). The great majority of insurance policies are paid for, or subsidised, by employers and thus insurance coverage is higher in the most prosperous, high-wage regions of the country. Even 35 years ago, when survey data were first gathered on this, insurance coverage in the south east of England (excluding London) was approximately 15%, whereas in northern England, Wales and Scotland the figure was 3–4%, which is at least a fourfold disparity. More recent data providing a geographical breakdown of insurance coverage are unfortunately not available from national social surveys. Nevertheless, we would expect NHS trusts located in the most prosperous regions of England to attract a higher level of private patient income from UK residents on the basis that household incomes are higher and the proportions of the population with private health insurance are also larger.

Fees from international patients have been an important source of income for NHS hospitals and UK private providers of healthcare; studies from the 1980s emphasised the significance of overseas patients to private institutions in London (Rayner, 1986; 1987) and their conclusions are echoed in more recent work by Lunt et al. (2015). We know that private patient income in the NHS in England rose tenfold in cash terms from the early 1990s to 2003–2004, reaching a total of some £370 million; after that point, the accounts of NHS foundation trusts were not consolidated but estimates drawn from Freedom of Information requests put the figures for 2016 at just under £600 million, suggesting continued growth. However, thus far, there is a lack of empirical work examining the distribution of private patient income at the level of individual trusts.

In substantive terms, therefore, we would anticipate differences in the charitable funding and private patient revenues in NHS acute trusts according to the level of local area deprivation. Finding lower levels of charitable support and private income for NHS trusts in more deprived areas would support institutional resources theories of ‘local area effects’, which propose that local area differences in organizational resources are a feature of inequality in individuals’ residential environments. We hypothesize that the extent to which trusts are able to draw upon such resources will therefore be negatively related to the degree of disadvantage of the patient population attending different trusts.

## Empirical approach: linking voluntary and private patient income to locational data on institutional beneficiaries

There are good reasons to suggest that, compared to less deprived areas, communities experiencing greater levels of disadvantage will be less able to financially support local institutions through charitable donations and private fees. However, testing this key hypothesis empirically poses the challenge of developing an appropriate measurement of location: when comparing the income of institutions in more deprived and less deprived areas, to what address should covariate data on deprivation be linked? The address of an organization provides one straightforward measure, which has been used in studies of charitable support for schools by Body et al. (2017) to identify the level of deprivation in the neighbourhoods surrounding schools. Other possibilities are to map the locations of the addresses of charitable donors or of the institutional beneficiaries. We examine each in turn.

First, an established stream of nonprofit and voluntary research examines spatial variation in voluntary activity by comparing areas using the addresses of voluntary organizations (Wolch & Geiger, 1983; Bielefeld et al., 1997; Fyfe & Milligan, 2003; Joassart-Marcelli & Wolch, 2003; Clifford, 2012; Clifford, 2018). The use of regulatory register data on the recorded address of these entities may not accurately capture spatial aspects of the operations of a non-profit entity which may be operating over multiple sites or, conversely, may be focussing its efforts on a very small part of a community (McDougle, 2015). Where available, it is therefore preferable to use the institutional ‘area of operation’—the local area where the organization ‘does its work or provides its benefit’ (Clifford, 2018).

Second, it is possible to examine spatial variation in charitable giving by comparing across areas based on the addresses of charitable donors. Unfortunately, the only reliable data available are from national surveys of individuals, which permit limited spatial disaggregation. Furthermore such data are based on the residential locations of respondents and ‘so it is impossible to state whether those who are … giving money to charity … are doing so in their own [local area]’ (Mohan & Bulloch, 2012, p. 13).

Third, we therefore propose that a preferable approach to measuring location, when comparing the income of institutions in more deprived and less deprived areas, is to use the addresses of the institutional beneficiaries. This approach is aligned to our objective of assessing whether local area differences in organizational resources are a feature of inequality in individuals’ residential environments. We characterize the levels of disadvantage in communities served by institutions using the addresses of their beneficiaries to produce an aggregate measure of the proportion of the population served by the institution and living in communities at particular points in the distribution of deprivation. We are not aware of any previous research in any country that has yet been able to systematically compare differences in public institutions’ charitable and private fee income according to differences in the local area characteristics of the communities served by these institutions—for the full distribution of local area contexts across a country.

### Data and method

We focused on the population of 117 acute non-specialist NHS trusts. We did not consider ambulance/community/mental health/specialist trusts, which are examined in a complementary paper (Bowles et al., 2023). We also did not consider primary care. To facilitate our analysis we linked data from a variety of different sources:

- Data on the total annual income of NHS trusts (calculated as: Operating income from patient care activities [SCI0100A] + Other operating income [SCI0110A]), and on the private patient income of NHS trusts, was from NHS England and NHS Improvement Trust Accounts Consolidation (TAC) data.
- Data on the total charitable income of the NHS charity linked to each NHS trust was from the Charity Commission’s Register of Charities (RoC). The RoC data, originally provided through the annual returns that charities are required to file as part of the Charity Commission’s regulatory process, contains key information on the activity of all registered charities in England and Wales. (Since charitable income is likely to be affected by temporary fluctuations, if the date was available, we used a three-year moving average of trusts’ charitable income: for example average annual charitable income for the financial years ending 2018, 2019 and 2020 at the end of the analysis period. We did not apply this process to NHS trusts’ total income or private patient income.)
- Data on the residential context of the beneficiaries of each NHS trust was from the Hospital Episode Statistics (HES) product published by NHS England, which groups provider spells by quintile of the Index of Multiple Deprivation (IMD) based on the postcode of the patient’s address for each non-specialist acute NHS trust. (Note that HES data does not include all mental health specific hospital activity and does not consider community and primary care.) The IMD is a summary measure based on the aggregation of individual characteristics in seven domains of deprivation relating to income; employment; health and disability; education, skills and training; barriers to housing and services; crime; and characteristics of the local environment (see Noble et al., 2006). The IMD is measured at the level of the lower super output area (LSOA), which has an average population of about 1,500 people, designed for the reporting of information about small areas. There are approximately 32,000 LSOAs in England.
- We identified teaching hospital trusts from the list of NHS trusts through linking to the list of members of the University Hospitals Association (2022). Teaching hospital trusts, usually affiliated with a university, provide health professionals with medical education.

In our analysis we began by considering the dependent variable *y*, the proportion of total NHS trust income that comes from charitable income. This proportion was observed in the interval [0, 1]. Therefore we used a fractional regression model, a generalized linear model with a binomial distribution and a logit link function (Papke & Wooldridge, 1996):

$$E(y|x)=\exp(x^{\prime }\beta )/(1+\exp(x^{\prime }\beta ))$$

where β is a vector of parameters and ***x*** is a vector of covariates. In our first model our only covariate was the key variable of interest measuring the context of the trusts’ beneficiaries: the percentage of hospital spells that are from the most deprived quintile of the IMD distribution. Our second model included controls for the size of the trust (measured by its total income) and whether the trust had teaching hospital status. Note that, of the 117 non-specialist acute trusts that we considered, 11 (9%) had total annual income less than £250 million; 57 (49%) had incomes between £250 million and £500 million; 37 (31%) had incomes between £500 million and £1 billion; and 12 (10%) had an income over £1 billion.

We then repeated the analysis above for different dependent variables *y*: the proportion of total NHS trust income that comes from private patient income; and the proportion of total NHS trust income that comes from charitable sources or private patient income.

## Results

**Figure 1.**
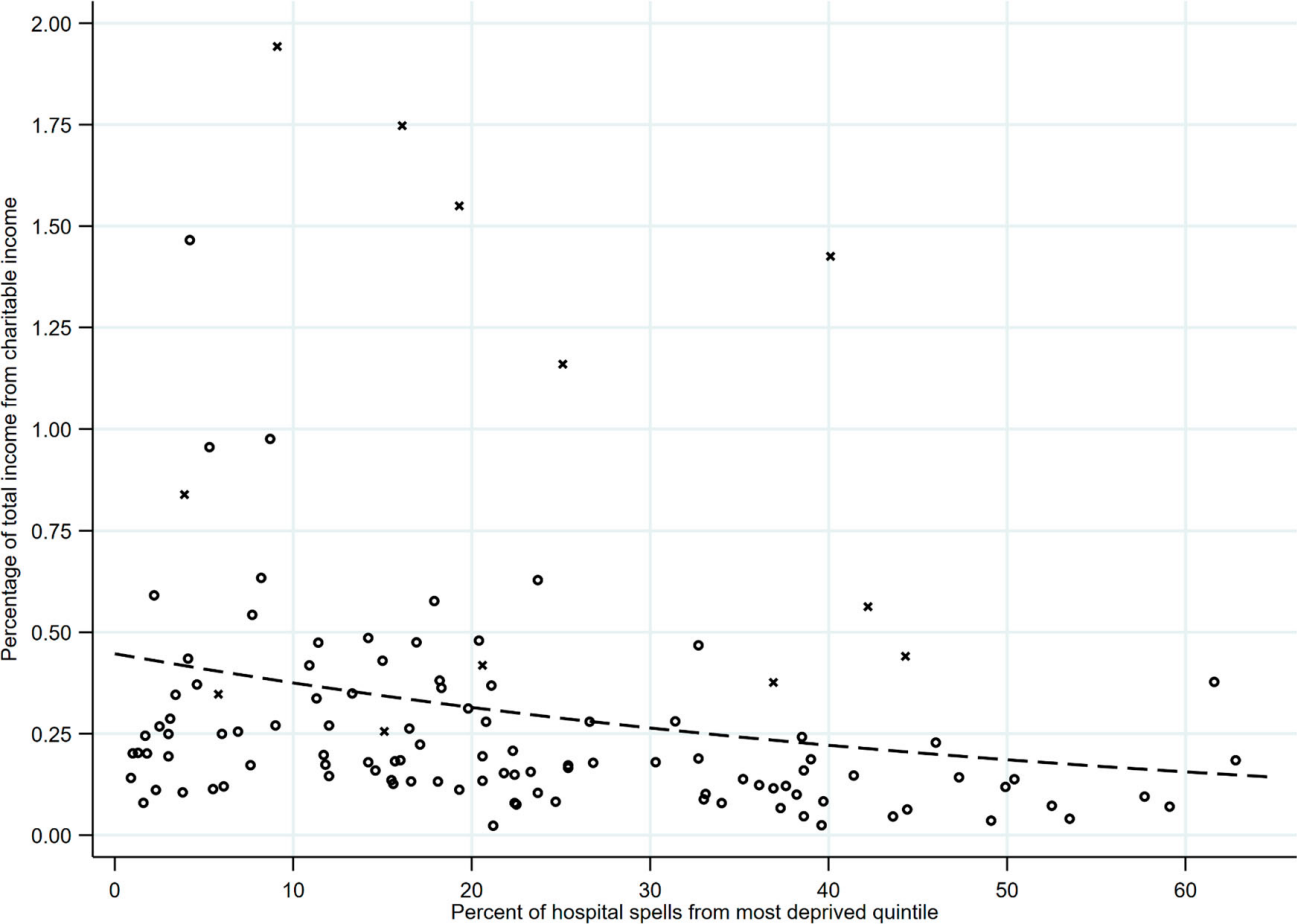
Relationship between deprivation and the percentage of total trust income from charitable income. *Notes: N* = 117 trusts. Circles are trusts with below £1 billion in annual income; crosses are trusts with above £1 billion in annual income. Dashed line provides fitted values, by deprivation, of the average percentage of total trust income from charitable income (based on Model 1 in Table 1).

**Figure 2.**
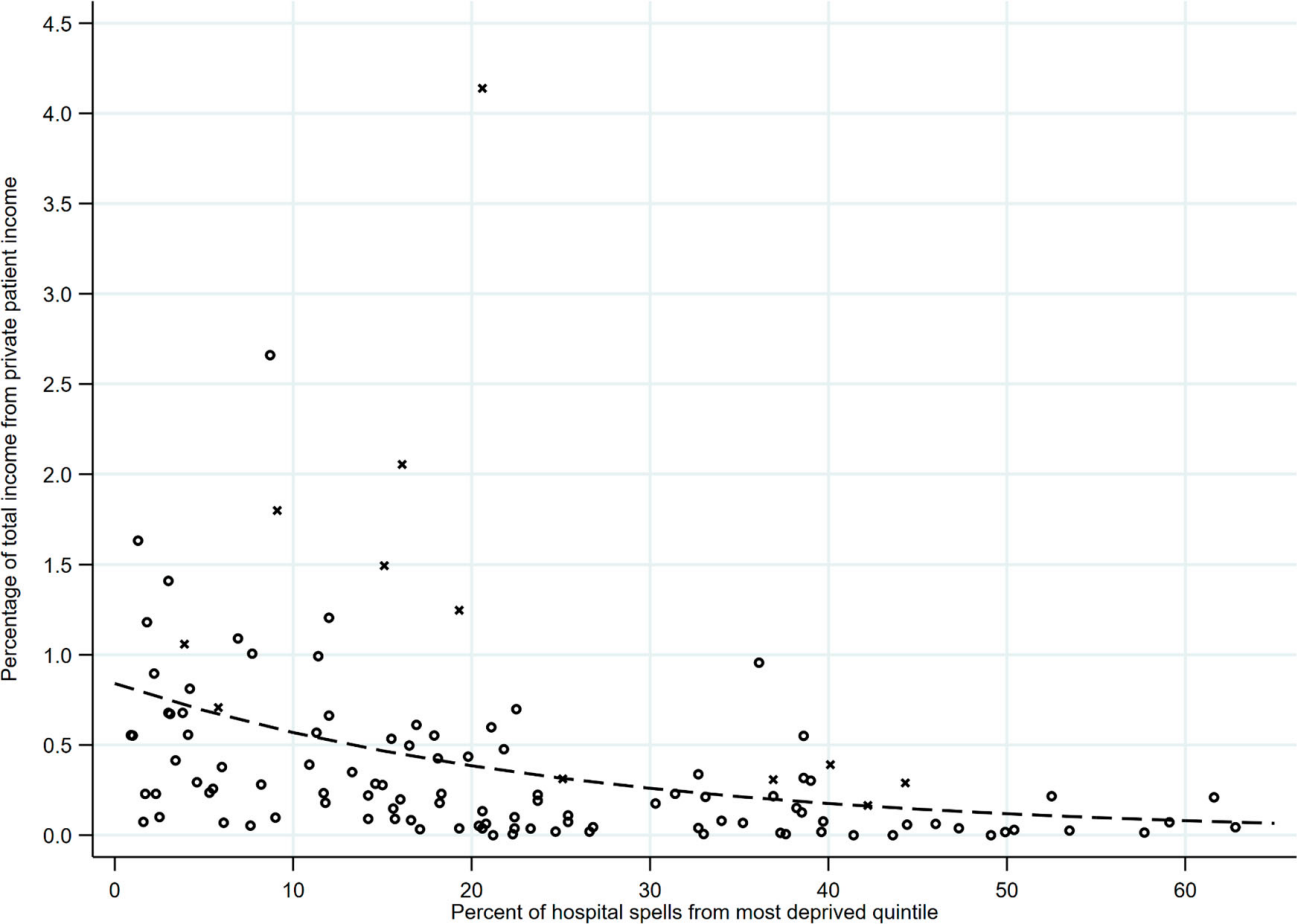
Relationship between deprivation and the percentage of total trust income from private patient income. *Notes: N* = 117 trusts. Circles are trusts with below £1 billion in annual income; crosses are trusts with above £1 billion in annual income. Dashed line provides fitted values, by deprivation, of the average percentage of total trust income from private patient income (based on Model 3 in Table 1).

**Figure 3.**
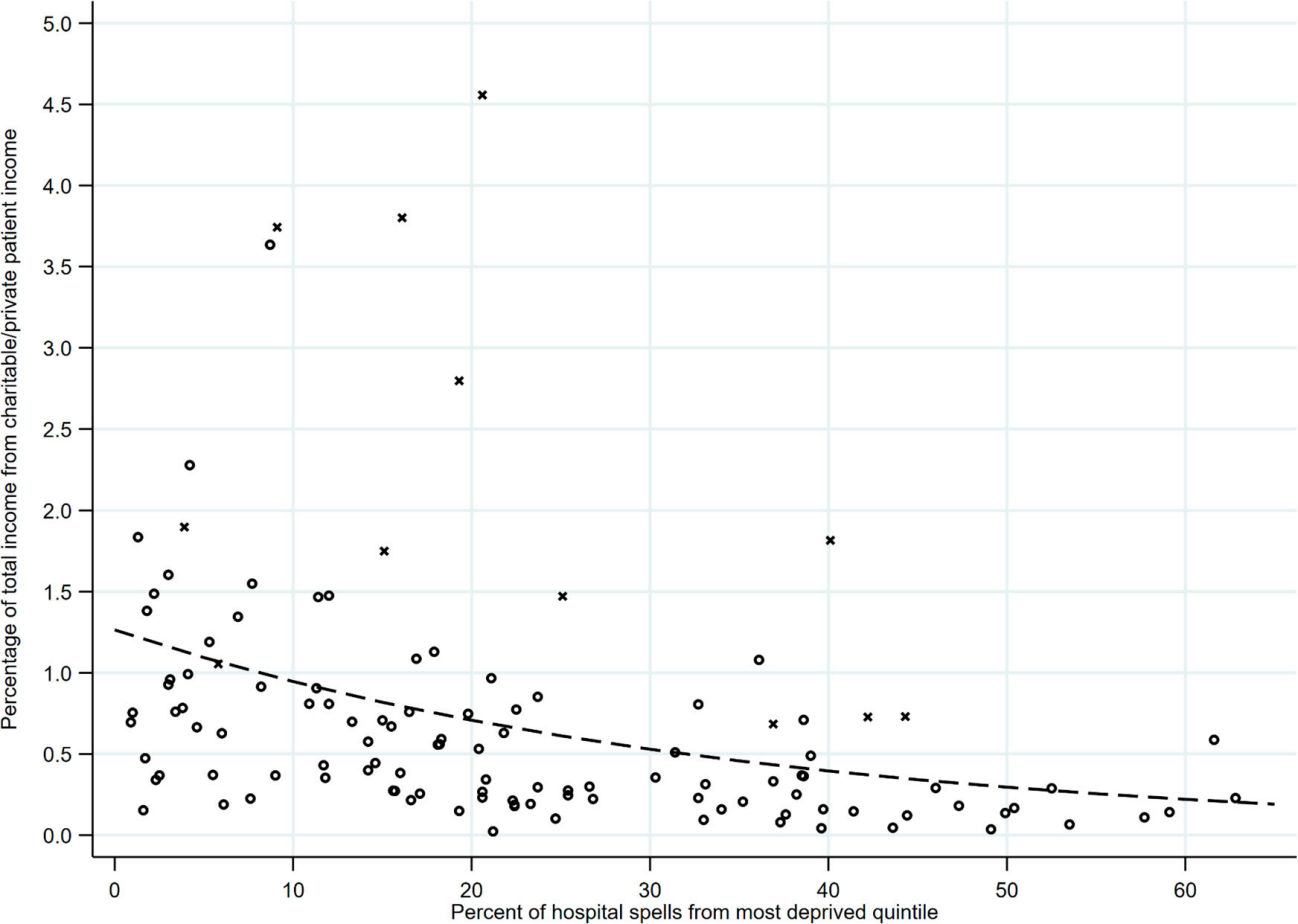
Relationship between deprivation and the percentage of total trust income from charitable/private patient income. *Notes: N* = 117 trusts. Circles are trusts with below £1billion in annual income; crosses are trusts with above £1 billion in annual income. Dashed line provides fitted values, by deprivation, of the average percentage of total trust income from charitable/private patient income (based on Model 5 in Table 1).

We examined the relationship between deprivation (in terms of the percentage of NHS trust hospital spells from the most deprived quintile of the IMD distribution) and each of our three outcome variables: the percentages of total NHS trust income that were, respectively, drawn from charitable sources (Figure 1), fees for treating private patients (Figure 2), and charitable sources and private fees combined (Figure 3). In summarizing the relationship between deprivation and these outcome variables, there are three particular aspects of the results to note.

First, there was a clear relationship between deprivation and the average of each of our outcome variables. As Model 1 (Table 1) summarizes, an increase in the percentage of hospital spells that are from the most deprived quintile of the IMD distribution is associated with a decrease in the average percentage of total NHS trust income that comes from charitable income (coefficient: -0.018; *p < *0.01). This relationship from Model 1 is illustrated by the dashed line in Figure 1, which provides the average percentage of total trust income from charitable income for different levels of deprivation. Where 5% of hospital spells were from the most deprived quintile of the distribution, an average of 0.41% (95% confidence interval [CI] 0.30–0.52) of total trust income was from charitable income. In contrast, where 60% of hospital spells were from the most deprived quintile of the distribution, an average of 0.16% (95% CI 0.07–0.24) of total trust income was from charitable income. Therefore the average percentage of total trust income from charitable income is 2.6 times higher (95% CI 0.9–4.3) in NHS trusts serving less deprived compared to more deprived communities. Note that, when we control for the size of trust and whether the trust has teaching hospital status (Model 2), the negative relationship between deprivation and proportion of income from charitable sources persists and, indeed, further strengthens (coefficient: -0.020; *p < *0.01). This is evidence that the differences in charitable income according to deprivation are not simply a reflection of differences in the type of NHS trust between more and less deprived areas.

**Table 1.** Fractional regression model coefficients (outcome: proportion of total trust income from given source).

Model No.	1	2	3	4	5	6
Income source	Charitable income		Private patient income		Charitable/private patient income	
						
Percentage of hospital spells from most deprived quintile	−0.018	−0.020	−0.039	−0.044	−0.029	−0.033
	(−2.92)**	(−3.80)***	(−6.13)***	(−5.98)***	(−5.80)***	(−6.43)***
Size (£ total annual income; Ref: <250 million)						
						
£250 million to 500 million		−0.015		0.246		0.138
		(−0.07)		(0.71)		(0.63)
£500 million to 1 billion		−0.049		0.264		0.130
		(−0.23)		(0.71)		(0.55)
£1 billion+		1.095		1.319		1.232
		(3.65)***		(2.79)**		(3.96)***
Status (Ref: not a teaching hospital trust)						
						
Teaching hospital trust		0.346		0.366		0.355
		(2.17)*		(1.49)		(2.00)*
LR χ2	8.498	65.007	37.559	69.948	33.694	85.047
df	1	5	1	5	1	5

*Notes:* Exponentiated coefficients. z statistics in parentheses. *N* = 117 trusts.

* *p* < 0.05, ** *p* < 0.01, *** *p* < 0.001.

As Model 3 (Table 1) summarizes, an increase in the percentage of hospital spells that are from the most deprived quintile of the IMD distribution is associated with a decrease in the average percentage of total NHS trust income that comes from private patient income (coefficient: -0.039; *p < *0.01; relationship summarized by the dashed line in Figure 2). Where 5% of hospital spells come from the most deprived quintile of the distribution, an average of 0.69% (95% CI 0.52–0.87) of total trust income comes from private patient income. In contrast, where 60% of hospital spells come from the most deprived quintile of the distribution, an average of 0.08% (95% CI 0.02–0.13) of total trust income comes from private patient income. Therefore the average percentage of total trust income from private patient income is 8.7 times higher (95% CI 2.7–14.7) in NHS trusts serving less deprived compared to more deprived communities. As before, when we control for the size of the trust, and whether the trust has teaching hospital status (Model 4), the negative relationship between deprivation and proportion of private patient income persists (coefficient: -.044; *p < *0.01). As with charitable resources, then, trust-level differences in private patient income are not simply a reflection of differences in the type of NHS trust between more and less deprived areas.

Second, while there is a clear relationship between deprivation and the *average* of each of our outcome variables, there is also residual variation around this average (Figures 1–3). Note that much of this residual variation is accounted for by outlying values, with a small number of trusts with distinctively high proportions of their income from charitable income/private patient income/charitable or private patient income. Importantly, many of these outlying values are trusts that are very large in size. We show this in Figures 1–3 by labelling with a cross, rather than a circle, those trusts with above £1 billion in total annual income. Notably many of the outlying trusts are large teaching hospital trusts in London. (Note that the differences in charitable income, and in private patient income, according to the size and status of trust are not the primary focus of this article but are evident in Table 1: compared to smaller trusts, very large trusts with an income of more than £1 billion have higher proportions of total trust income from charitable income [Model 2] and from private patient income [Model 4].)

However, there is also residual variation around the average above and beyond that represented by the outlying values. Note that this variation about the average is most marked at low values of deprivation. Where a high percentage of NHS trust hospital spells are from the most deprived quintile of the IMD distribution, there is low variation, with most trusts receiving relatively low levels of charitable income or private patient income as a proportion of their total income (Figures 1–3). However, where a low percentage of NHS trust hospital spells are from the most deprived quintile of the IMD distribution, there is more variation—with some trusts receiving relatively low levels of charitable income or private patient income as a proportion of their total income and some receiving relatively high levels. This suggests that lack of deprivation is a necessary but not sufficient condition for trusts to receive higher proportions of their income from charitable sources or private patient fees.

Third, the differences according to deprivation in charitable income and private patient income reinforce one another since they act in the same direction. Indeed, where we consider our third outcome, the proportion of total NHS trust income that is accounted for by *either* charitable income *or* private patient income, the differences according to deprivation are accentuated in absolute terms (Model 5, Table 1; relationship summarized by the dashed line in Figure 3). Thus, where 5% of hospital spells come from the most deprived quintile of the distribution, an average of 1.10% (95% CI 0.85–1.33) of total trust income comes from either charitable income or private patient income; where 60% of hospital spells come from the most deprived quintile of the distribution, an average of 0.22% (95% CI 0.12–0.32) of total trust income comes from either charitable income or private patient income. (As before, when we control for the size of trust and whether the trust has teaching hospital status [Model 6], the negative relationship with deprivation persists [coefficient: -0.033; *p *< 0.01], suggesting that the differences in private patient income or charitable income are not simply a reflection of differences in the type of hospital between more and less deprived areas.) Therefore the absolute difference between more and less deprived areas is most sizeable when we consider charitable income and private patient income in combination (absolute difference of 0.9% of total NHS trust income; 95% CI 0.6–0.12), rather than when we consider charitable income in isolation (absolute difference of 0.3% of total NHS trust income; 95% CI 0.1–0.4), or when we consider private patient income in isolation (absolute difference of 0.6% of total NHS trust income; 95% CI 0.4–0.8).

## Discussion

This article has provided the first analysis of inequalities by area deprivation in the charitable income and private patient income of the population of acute non-specialist NHS trusts in England. The analysis was built on a unique dataset which links TAC data on total trust income and private patient trust income, Charity Commission data on the charitable income of NHS charities associated with each trust and HES data on the residential context of the beneficiaries of each trust. To our knowledge this is an entirely novel study, in a UK context, which provides valuable empirical evidence of spatial inequality in the private resources that support local public institutions.

Compared to NHS trusts serving less deprived communities, NHS trusts serving more deprived communities on average receive a much lower proportion of their total income from both charitable income and from private patient income. These results are consistent with theory about the spatial manifestations of resource insufficiency, which predicts that, given well-established research which shows that individuals with higher financial resources and higher levels of education give more in absolute terms to charitable causes, philanthropic financial resources are likely to vary significantly according to the compositional socioeconomic characteristics of local areas (Salamon, 1987; Reich, 2018). We also show that local areas differ not only in the opportunity for trusts to benefit from charitable donations—but also in the opportunity for trusts to receive income from private patients. Therefore these spatial inequalities are reinforcing: on average, NHS trusts in more deprived local areas are *doubly disadvantaged* through a lower level of both charitable income and private patient income.

Therefore our results are relevant to institutional resources theories of ‘local area effects’ which propose that local area differences in organizational resources may be a feature of inequality in individuals’ residential environments (Galster, 2012; Clifford, 2018). In particular they suggest that the resources for public service provision can be affected by area disadvantage even in the European welfare state context where the redistributive welfare programmes ensure that a significant public institutional infrastructure is maintained in areas of disadvantage (Hastings, 2009, p. 509).

The post 1980 history of the NHS has seen two periods of severe resource shortages—from 1980 to the early 1990s and from 2010 onwards—placing considerable pressure on the budgets of service providers. In this context, successive governments have sought to pluralize and diversify the funding base of the service. However, as we show in this article, a consequence has been that some NHS trusts have benefited to a greater extent than others from charitable and private patient income. We note that, overall, the proportion of total NHS trust income accounted for by charitable income and private patient income is small (Figures 1–3). Why might spatial variation in this proportion be an issue of concern to policy-makers given that charitable donations and private patient fees might be regarded as a private matter? In particular, ‘freedom from the canons of social justice’ (Simey, 1937, p. 133) might be considered intrinsic to the character of voluntary action: resources will not always be allocated in accordance with abstract criteria such as need or equity. Some writers therefore argue that it is inappropriate to compare the imperfect distribution of charitable resources with an idealized vision of the equitable distribution of the public sector (Prochaska, 1992, p. 130). In any case, given the limited aggregate contribution of charitable funds and private patient income to the NHS, it would be hard to argue that these sources of funds are totally incompatible with the egalitarian aims of the health service.

On the other hand, the positive impact of charitable and private patient income may be organizational as well as financial. For example, treating private patients may add status to a hospital and enable it to attract and retain high-quality staff, in particular consultants (Walpole, 2019, p. 7). Furthermore, though the financial sums involved may appear relatively small compared to the totality of the NHS budget, the tight funding constraints on the service, especially since the UK’s 2010 general election, mean that for individual NHS agencies, ‘even apparently marginal additions of financial resource may be of considerable value’ (Bowles et al., 2023, p. 2). From this perspective, the uneven distribution of charitable and private patient income across NHS trusts would have implications for equity if it meant that trusts had differential access to capital or different levels of funds to improve patient welfare, especially ‘if the availability of charitable funds were to influence the trajectory of [trust] development’ (Mohan, 2002, p. 200; see also Bowles et al., 2023). These themes are of particular policy salience given the emphasis placed on addressing spatial inequalities by the UK government since 2019, when the Conservative party was re-elected on a programme which made commitments to ‘levelling up’ inequalities between communities (see also Kruger, 2020). Discussions of relevant policy initiatives has often referenced the role that might be played by voluntary action and voluntary organizations in economic and social renewal.

However, our research suggests that such efforts will widen, not narrow, differentials between communities. In a context of severe restrictions on the growth of public funding, this provides further support for the argument (Gibbons & Hilber, 2022) that charitable giving was not stimulated in response to post 2010 reductions in public expenditure. These themes may become even more salient given the focus on increasing the amount of commercial income within the current NHS Long Term plan. NHS trusts across England are being encouraged by NHS England/Improvement (NHSE/I) to ‘actively explore and develop opportunities to … grow their external (non-NHS) income’ (NHS, 2022, para. 151; see also Exworthy et al., 2023; Exworthy & Lafond, 2021), with a focus on supporting the development of ‘private patient opportunities to generate revenue’ (see Housden, 2022). Meanwhile—with prominent charitable fundraising campaigns under way aimed at raising significant sums for NHS capital developments—the place of charity in the NHS also seems likely to be an issue of growing significance in the coming years. In this substantive context the lack of previous research examining spatial variations in the charitable and private patient income of NHS trusts is a serious omission.

Further research on these themes—and in particular research which is able to shed light on the reasons underlying the residual variation in funding between NHS trusts, such that lack of deprivation is a necessary but not sufficient condition to receive higher proportions of income from charitable or private patient income—is a priority.

There is also a need to open up discussions about how best to mitigate spatial differences in the charitable and private patient income of NHS trusts, particularly if these sources of income grow in importance going forward. From one perspective, charitable donations may be regarded as a private matter for donors and recipients, which cannot readily be influenced by public policy (Bowles et al., 2023). Indeed, donations may be motivated by geographical proximity and personal ties, rather than being necessarily informed by an assessment of relative healthcare need (Lattimer et al., 1996; Mohan & Breeze, 2016). Nevertheless, it may be appropriate to be sensitive to the spatial distribution of charitable income received by individual NHS trusts when considering how best to distribute charitable income received by the NHS through national—rather than local—fundraising, such as through NHS Charities Together (the national organization of the principal NHS charities). In any case, if effective responses are to be developed to spatial differences in the private financial resources that support the NHS, continuing to monitor both the charitable and private patient income of individual NHS trusts is a priority for future empirical work.
